# Adipose tissue is a predictor of 30-days mortality in patients with bloodstream infection caused by carbapenem-resistant *Klebsiella pneumoniae*

**DOI:** 10.1186/s12879-022-07108-9

**Published:** 2022-02-21

**Authors:** Piaopiao Ying, Jiajing Chen, Yinchai Ye, Jianzhong Ye, Weiyang Cai

**Affiliations:** 1 Department of General Medicine, The Affiliated Hospital of Hangzhou Normal University, Hangzhou, China; 2 Department of Pneumology, The First Affiliated Hospital of Wenzhou Medical University, Wenzhou, China; 3 Department of General Medicine, The Health Center of Eryuan Town, Wencheng County, Wenzhou, China; 4 Department of Clinical Laboratory, The First Affiliated Hospital of Wenzhou Medical University, Wenzhou, 325000 China; 5 Department of Gastroenterology, The First Affiliated Hospital of Wenzhou Medical University, Wenzhou, 325000 China

**Keywords:** Adipose tissue, Carbapenem-resistant *Klebsiella pneumoniae*, Bloodstream infection, 30-Day mortality, Nomogram

## Abstract

**Background:**

Prevalence of carbapenem-resistant *Klebsiella pneumoniae *(CRKP) bloodstream infection with high mortality has attached physicians' attention. High visceral adipose tissue (VAT) and high subcutaneous adipose tissue (SAT) were confirmed by previous studies that were closely related to increased pneumonia severity, more complications, and higher mortality in COVID-19. Thus, we speculate that CT-quantified body composition may also be connected to all-cause mortality and bacterial clearance in patients with CRKP bloodstream infection (BSI).

**Methods:**

We investigated the associations of CT-quantified body composition with the mortality of CRKP bloodstream infectious patients. All CT images were obtained at the level of the L3/4 spinal level. The prognostic value of the body composition was analyzed using the Cox regression model, and precise clinical nomograms were established.

**Results:**

72 eligible patients both suffered from CRKP bloodstream infection and performed abdominopelvic CT were included. Factors associated with 30-day all-in hospital mortality included total adipose tissue (TAT) [adjusted hazard ratio (HR) = 1.028, 95% confidence interval (CI), 1.003–1.053; P = 0.025], age [HR = 1.030, 95% CI, 1.000–1.061; P = 0.047] and SOFA scores [HR = 1.138, 95% CI 1.049–1.263; P = 0.002]. Compared with low-VAT, patients with high-VAT show a strikingly poor prognosis in both 30-day all-cause mortality (P = 0.0108, Fig. 2A) and 30-day CRKP BSI mortality (P = 0.0049, Fig. 2C). The results of TAT were similar to VAT.

**Conclusions:**

Our study suggested that CT-derived body composition could be a credible and effective alternative to assess the prognosis of patients with BSI owing to CRKP. CT-quantified TAT, age, and SOFA scores were independently associated with 30-day all-cause mortality in these severe infectious patients, while skeletal muscle did not have obvious statistical significance.

## Background

Currently, the emergence of carbapenem-resistant *Klebsiella pneumoniae* (CRKP) is rapidly increasing with the growing usage of carbapenems, posing a severe threat to vulnerable patients and augmenting the burden on the public health system [1–3]. Furthermore, the mortality of CRKP bloodstream infection patients ranged from 33.0 to 52.8% in 28- or 30-day all-cause mortality, arousing global attention [4, 5]. Therefore, it is crucial to identify the prognostic factors of 30-day or all-cause mortality in the early time and take effective and targeted intervention measures to reduce mortality due to CRKP bloodstream infection (BSI) [6]. According to previous literature, overweight and obesity were found associated with influenza A and Coronavirus Disease 19 (COVID-19) complications, severity, and mortality [7–9]. Traditional index, such as body mass index (BMI), however, is insufficient for reflecting the distinctions between fat and muscle mass, or visceral adipose tissue (VAT), subcutaneous adipose tissue (SAT), and skeletal muscle (SM). Abdominopelvic computed tomography (CT) imaging is not only a routine examination that assists clinical management with the diagnosis of critically ill patients but also a more accurate method to differentiate body composition. Moreover, CT-defined body composition is widely confirmed for accurately reflecting on different types of adipose tissue as well as muscle mass.

High-VAT and high-SAT were confirmed by previous studies that are closely related to increased pneumonia severity, more complications, and higher mortality in COVID-19 [10, 11], and a pioneering study had revealed the association of visceral adipose tissue (VAT), subcutaneous adipose tissue (SAT) and infections caused by *Mycobacterium avium* [7]. Thus, we speculated that CT-quantified body composition may also be connected with all-cause mortality and bacterial clearance in patients with CRKP BSI. Besides, it is not suitable for severely infected patients to weigh them with the conventional methods so that data on the weight of those who were missing during their hospitalization. CT-defined body components provided an alternative for clinical physicians to acquire and assess the nutritional status of extremely severe infectious patients and develop a personalized treatment plan for them.

Therefore, the primary aim of our study was to explore the relationship between CT-quantified body composition (VAT, SAT, and SM) and 30-day mortality in patients with BSI owing to CRKP. Additionally, the independent risk factors in these patients were analyzed. Furthermore, we tried to construct the 30-day mortality nomogram to predict 14-day or 30-day survival probability based on the prognostic factors derived from multivariate Cox regression analysis.

## Methods

### Study population and design

This study was a single-center retrospective cohort research, approved by the ethics committee of the First Affiliated Hospital of Wenzhou Medical University, one of the largest health care centers in the southern province of Zhejiang, China. Patients, both suffered from CRKP BSI supported by clinical symptoms and etiological evidence during their hospitalization and performed abdominopelvic computed tomography imaging from 2016 January 1st to 2020 June 1st, were eligible for the study. CRKP BSI were nosocomial infections and was first detected during hospitalization in our hospital. Meanwhile, Abdominopelvic CT examinations were performed in these infectious patients due to abdominal trauma, enteric or urinary tract infection, severe sepsis, multiple organ dysfunction syndromes, etc. Patients who did not detect CRKP BSI for the first time in our hospital, less than 16 years old, or refused further treatment were excluded. CRKP was defined as minimum inhibitory concentration (MIC) ≥ 4 mg/L to both imipenem and meropenem according to Clinical and Laboratory Standards Institute guidelines [12].

### Variables and definitions

Patients' baseline data, including demographic characteristics, presence of comorbid conditions, acute complications (shock, acute respiratory failure, acute renal failure), Sequential Organ Failure Assessment (SOFA) Scores, exposure to invasive intervention surgery, renal replacement therapy, infection site, combined viral and fungal infection, Pitt bacteremia scores, and details on therapy were reviewed and obtained from electronic medical records. The observational onset of our research was defined as the date of microbiology specimen collection in the first cultivated CRKP. The date: SOFA scores, body temperature, and inflammation indicators of blood were recorded within 48 h before positive blood culture with CRKP infection. Antibiotic treatment, exposure to tracheostomy and mechanical ventilation, renal replacement therapy, and acute complications (respiratory failure, kidney failure, and shock) maintained for more than 48 h were considered for analysis. Surgery was defined as invasive procedures which could be found in the surgical record in the electronic medical records within 1 month before and after the CRKP BSI. Cultivated fungal, detection of 1,3-β-d-glucan or galactomannan antigen-positive, combined with the patient’s symptoms, signs, and antifungal drug treatment was referred to as fungal infection. Equally, the related viral nucleic acid tests and the clinical conditions of infectious patients were treated as viral infections. The average Chinese weight, 66 kg for males and 57 kg for females, was an alternative to some of these seriously infected patients in case of lacking weight records. Based on the recommended medication and antimicrobial susceptibility test of *Klebsiella pneumoniae,* antibiotic treatment options were divided into polymyxin B-based (PMB-based) or tigecycline-based (TGC-based) combination therapy or others (aminoglycosides, Fosfomycin, carbapenem, etc.) [13]. Early appropriate antibiotic therapy was the regard of administered 48 h or less by the prescribing physicians after the first culture of CRKP and included at least two in-vitro active drugs [14–16].

### Body composition

**Fig. 1:**
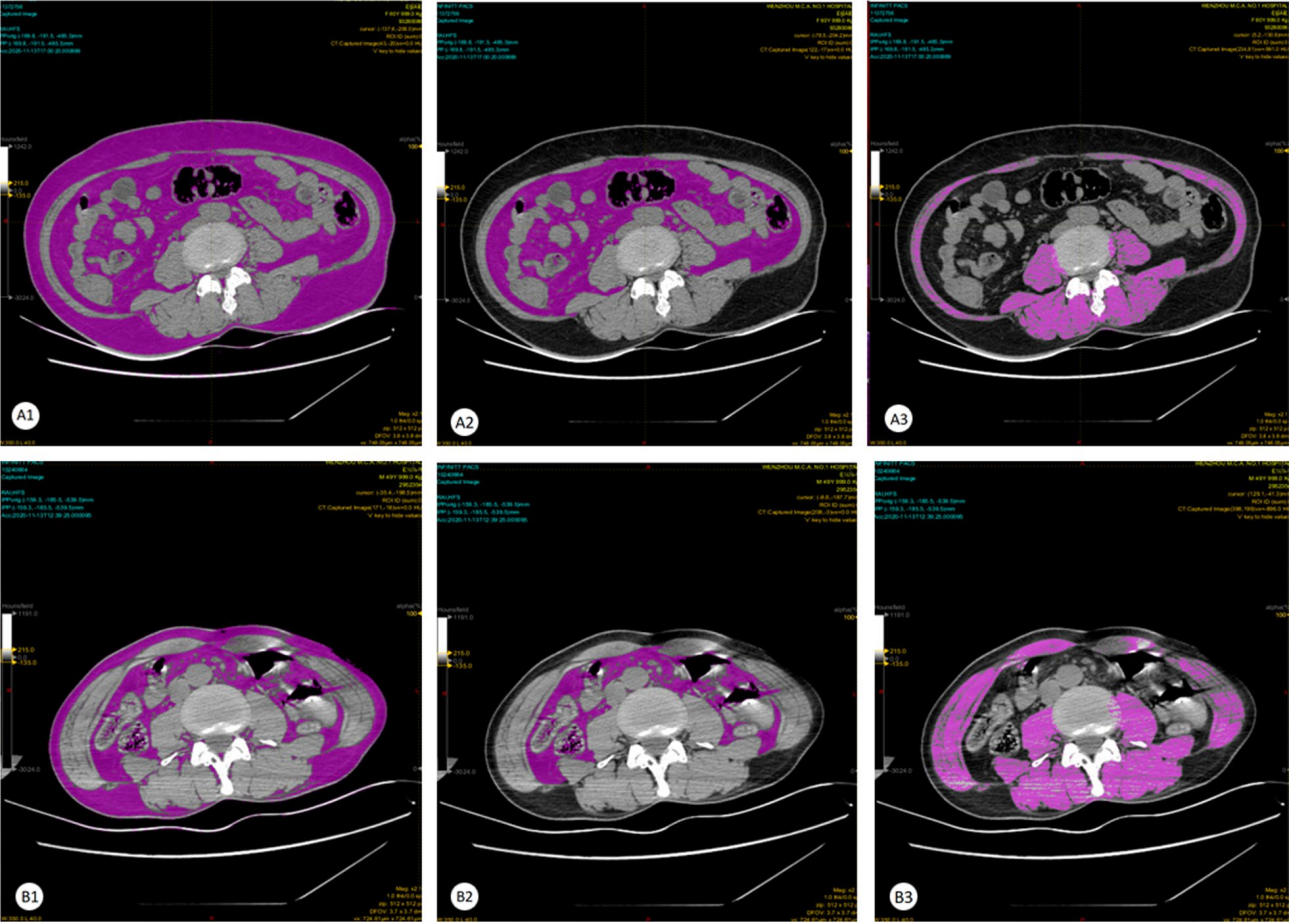
Example of a computed tomography (CT)-scan with the area-based, densitometric quantification of adipose tissue (threshold: − 190 to − 30 HU) measured at spinal level L3/4: region of interest (ROI) containing total adipose tissue (TAT) (**A1** and **B1**) and visceral adipose tissue (VAT) (**B1** and **B2**); and an example of the densitometric quantification of skeletal muscle (SM), dorsal and psoas muscles (threshold: 40 to 100 HU) (**C1** and **C2**)

Area-based quantifications of adipose tissue and muscle mass compartments were performed on the L3/4 spinal level using a semiautomatic software tool (Syngo Volume tool, Siemens Healthcare, Munich, Berlin, Germany) [17]. Two experienced radiologists drew the eligible CT planar. Records of multidetector-CT scans with quantification of body composition were retrieved 7 days before or after positive blood culture with CRKP. If the patient has undergone multiple abdominal CT examinations, the time point closest to the starting point of our experiment was chosen. The manually determined specific region of interest (ROI) includes VAT (the fascial plane of the abdominal muscle wall, using standard Hounsfield Unit (HU) range − 190 to − 30, Fig. 1) area expressed in mm^2^, TAT (whole abdominal circumference, using HU range − 190 to − 30), SM (sum of M. psoas major, M. erector spinae, M. quadratus lumborum, M. latissimus dorsi, M. transversus abdominis, M. obliquus internus abdominis and externus abdominis, and M. rectus abdominis, measured by limiting the attenuation threshold between 40 and 100 HU, Fig. 1) area expressed in mm^2^, the subcutaneous adipose tissue (SAT) area was defined as the subtraction between total adipose tissue (TAT) area and VAT area [18]. All CT examinations were performed using scanners: Brilliance-64, Philips Medical Systems, Eindhoven, The Netherlands; 128-MDCT scanner Somatom Definition, Siemens Health-care Sector, Forchheim, Germany.

### Statistical analyses

R software, GraphPad Prism, and Stats were conducted for statistical analyses. The 14-day and 30-day survival nomograms were constructed based on the prognostic factors derived from multivariate Cox regression analysis to predict 14-day and 30-day survival possibilities. Continuous variables were exhibited for means, medians, range, and standard deviation (SD) and compared using an independent t-test or Wilcoxon test; Spearman's correlation coefficient was used for variable correlation; Chi-square test was used to analyze categorical variables; log-rank survival analysis was employed to determine the effect of various variables on patients’ 30-day survival. The optimal cut-off values for VAT and TAT were calculated by the X-tile program (constructed by Yale University, New Haven, CT, USA). The cut-off of high TAT and VAT were 36.95 mm^2^ and 18.96 mm^2^, respectively. All statistical tests were two-sided and P < 0.05 was considered statistically significant.

## Results

### Participators characteristics

A total of 90 eligible patients, both suffered from CRKP bloodstream infection and had abdominopelvic computed tomography within 7 days were recruited from the First Affiliated Hospital of Wenzhou Medical University, a 4100-bed general teaching hospital; of these, 2 were excluded from the analysis due to incomplete information, 6 were excluded owing to cultivated CRKP before entering our hospital, 3 were excluded because of only 1 day of hospitalization, 7 were excluded for failed abdominopelvic CT image. Abdominopelvic CT examination was routinely implemented for these severe infectious patients with some complex or critical complications according to doctors' experience. As of discharge time, 27 patients died during 30 days follow-up, and the average survival time was 24.14 days. Additional baseline clinicopathological parameters were presented in Table 1.

**Table 1:** Demographic and clinical characteristics of CRKP bloodstream infectious patient’s univariate analysis of risk factors associated with survival

Demographic data	No. (%) or mean (SD)			P value
	Survival	Non-survival	All	
	n = 45	n = 27	n = 72	
Age (year)	60.18 (13.20)	70.30 (13.83)	63.97 (14.22)	0.003
Male	35 (77.8%)	25 (92.6%)	60 (83.30%)	0.190
Surgery	16 (35.6%)	6 (22.2%)	22 (30.6%)	0.234
Creatinine, umol/L	86.47 (108.87)	123.59 (134.35)	100.39 (119.51)	0.204
CRP, mg/L	71.33 (29.92)	79.96 (38.54)	74.57 (33.42)	0.292
PCT, ng/ml	6.09 (15.67)	11.14 (15.13)	7.98 (14.94)	0.166
SOFA score, points	5.84 (3.90)	9.70 (4.45)	7.32 (4.50)	0.000
Pitt bacteremia score, points	3.29 (2.74)	5.15 (3.29)	3.99 (3.07)	0.017
*Co-morbidities*				
Cardiovascular disease	1 (2.2%)	5 (18.5%)	6 (8.3%)	0.025
Chronical obstructive pulmonary disease	0 (0.0%)	1 (3.7%)	1 (1.4%)	0.375
Chronic kidney disease	3 (6.7%)	5 (18.5%)	8 (11.1%)	0.142
Diabetes mellitus	5 (11.1%)	10 (37.0%)	15 (20.8%)	0.009
Central nervous system disease	5 (11.1%)	5 (18.5%)	10 (13.9%)	0.486
Cancer	7 (15.6%)	3 (11.1%)	10 (13.9%)	0.733
Charlson comorbidity index	1.29 (1.71)	2.22 (2.08)	1.64 (1.89)	0.042
*Acute complications*				
Shock	12 (26.7%)	12 (44.4%)	24 (33.3%)	0.121
Acute respiratory failure	12 (26.7%)	9 (33.3%)	21 (29.2%)	0.574
Acute renal failure	6 (13.3%)	8 (29.6%)	14 (19.4%)	0.091
Tracheal intubation status	12 (26.7%)	10 (37.0%)	22 (30.6%)	0.355
*Body compositions*				
Skeletal muscle	5.22 (2.65)	5.97 (3.30)	5.50 (2.91)	0.294
Visceral adipose tissue	11.03 (5.90)	15.37 (8.39)	12.66 (7.20)	0.012
Subcutaneous adipose tissue	11.67 (6.80)	14.93 (12.00)	12.89 (9.16)	0.145
Total adipose tissue	22.70 (11.07)	30.30 (16.56)	25.55 (13.79)	0.022
*Infection type*				
Catheter related infections	7 (15.6%)	3 (11.1%)	10 (13.9%)	0.733
Hydrothorax or ascites	4 (8.9%)	2 (7.4%)	6 (8.3%)	1.000
Pulmonary	17 (37.8%)	18 (66.7%)	35 (48.6%)	0.018
Abdominal	13 (28.9%)	7 (25.9%)	20 (27.8%)	0.786
Urinary	15 (33.3%)	5 (18.5%)	20 (27.8%)	0.174
Skin and soft tissue	4 (8.9%)	5 (18.5%)	9 (12.5%)	0.281
Combined viral infection	4 (8.9%)	2 (7.4%)	6 (8.3%)	1.000
Combined fungal infection	20 (44.4%)	16 (59.3%)	36 (50.0%)	0.224
*Details of antibiotics*				
Early appropriate therapy	28 (62.2%)	14 (51.9%)	42 (58.3%)	0.388
PMB-based therapy	10 (22.2%)	8 (29.6%)	18 (25.0%)	0.482
TGC-based therapy	22 (48.9%)	9 (33.3%)	31 (43.1%)	0.197
Other antibiotics therapy	13 (28.9%)	10 (37.0%)	23 (31.9%)	0.473

*CRP* C-reactive protein, *PCT* procalcitonin, *SOFA* sequential organ failure assessment, *PMB-based therapy* polymyxin B-based therapy, *TGC-based therapy* tigecycline-based (TGC-based) combination therapy

### Associations of body composition with survival

**Fig. 2:**
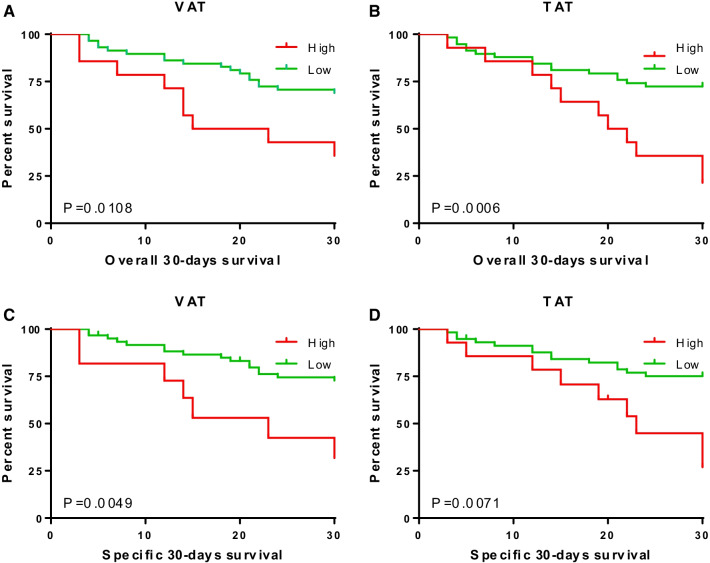
Outcomes of CRKP infection patients based on CT Body Composition. 30-day all-cause mortality based on visceral adipose tissue (**A**) and total adipose tissue (**B**). All-cause mortality based on visceral adipose tissue (**C**) and total adipose tissue (**D**)

As for the association of adipose fat and muscle tissue with 30-day mortality, we observed that TAT and VAT were dependently related to 30-day mortality while SM had no differences (Table 1). Furthermore, high-TAT was closely associated with worse clinical outcomes, after adjusting for comorbid conditions and other differences in baseline characteristics (Table 2). Compared with low-VAT, patients with high-VAT showed a strikingly poor prognosis in both 30-day all-cause mortality (P = 0.0108, Fig. 2A) and 30-day CRKP BSI mortality (P = 0.0049, Fig. 2C). The results of TAT were similar to VAT (Fig. 2B, D).

**Table 2:** Comparison between survival and non-survival using COX regression analysis

Risk factors	Univariate analysis	Multivariate analysis		
	P value	Adjusted HR	95% CI	P value
SOFA score	0.001	1.138	1.049,1.263	0.002
Age	0.004	1.030	1.000,1.061	0.047
Total adipose tissue	0.031	1.028	1.003,1.053	0.025

SOFA, sequential organ failure assessment

Cox regression was used for estimating the impact of these demographic and clinical characteristics on CRKP bloodstream infectious patient’s mortality outcomes for confounding variables based on P < 0.1 in univariate analysis

### Prognostic scores for survival

In the multivariable analysis (Table 2), factors associated with 30-day all-in hospital mortality included total adipose tissue (TAT) [adjusted hazard ratio (HR) = 1.028, 95% confidence interval (CI), 1.003–1.053; P = 0.025], age [HR = 1.030, 95% CI, 1.000–1.061; P = 0.047] and SOFA scores [HR = 1.138, 95% CI 1.049–1.263; P = 0.002].

**Fig. 3:**
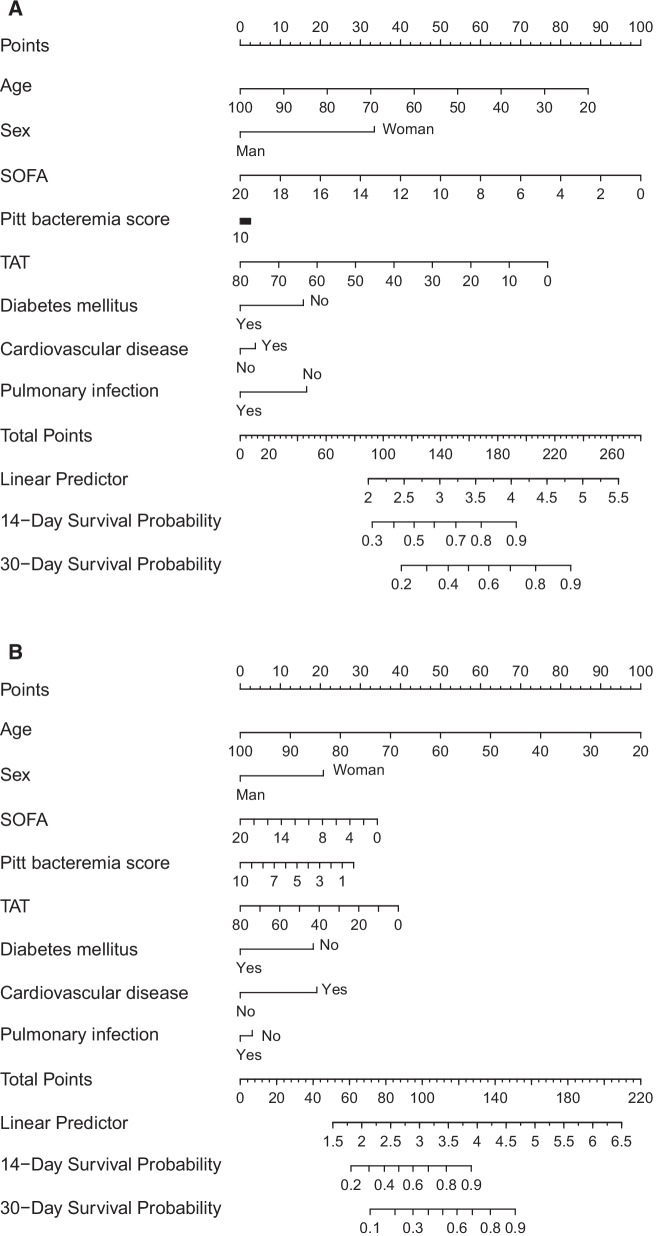
The nomogram to predict the 30-day all-cause mortality (**A**) and 30-day mortality owing to CRKP BSI (**B**) of CRKP bloodstream infectious patients

A nomogram was constructed to predict 14- and 30-day survival of patients with CRKP bloodstream infectious (Fig. 3). Total scores were summations of each variable based on the intersection of the vertical lines. As shown in Fig. 3, VAT, TAT, age, and SOFA scores contributed the most risk points (ranged 0–100), whereas the other clinical information contributed much less. By using a nomogram, we could precisely convert each patient's clinical index to the corresponding point, and then evaluate the likelihood of survival. HR values for therapy progression derived from Cox models suggested that patients with lower TAT are more likely to survive from CRKP bloodstream infection. The combination of body composition, age, and SOFA showed a good ability to predict survival.

## Discussion

The evaluation of body composition by abdominal CT imaging in CRKP BSI patients has not previously been reported. So far, to our knowledge, it was the first study to assess the correlation between CT-defined body composition and survival of CRKP BSI patients. Based on the Cox regression and nomogram of 14-day and 30-day mortality in the included patients, the main finding of our study was that high-TAT, age, and SOFA scores were associated with worse clinical outcomes, while skeletal muscle did not have obvious statistical significance.

Prevalence and high mortality among patients suffering from CRKP bloodstream infection have attached physicians' attention, especially these individuals with important morbidities. Hence, it was necessary for us to early identify the risk prognostic factors leading to the death of these patients and take targeted and effective intervention methods to reduce mortality. CT-quantified subcutaneous and visceral adipose tissue were identified as an extremely significant risk factor for COVID-19 patients with more severe complications and higher mortality based on the present proof-of studies [10, 19]. In addition, CT-derived body composition would be a credible and effective alternative to assess patients' nutritional status, especially for those severely infected patients lacking body weight to calculate BMI.

Univariate analysis showed that CRKP BSI patients who had higher visceral adipose tissue and total adipose tissue were more likely to die, while skeletal muscle had no predictive meaning, which was similar to the results of CT-defined body components on the prognosis of COVID-19 [11, 20]. It is generally acknowledged that more fat area is prone to develop metabolic diseases characterized by carbohydrate, lipid, and protein metabolic disturbances, resulting in insulin resistance, hyperglycemia, hyperlipidemia, hypoalbuminemia as well as their complications [21]. Meanwhile, as we did in the univariate analysis of the death group and the survival group, patients with cardiovascular disease or diabetes mellitus had a worse prognosis [22, 23]. Unfortunately, these metabolic-related morbidities often co-exist in a single individual, playing a significant role in the mortality of CRKP BSI patients. In addition, excessive adipose tissue, especially visceral adipose tissue, was strongly associated with systemic inflammatory status and the delay of the immune response in the pathophysiological pathways, recently highlighted in COVID-19. Patients with impaired immune response were likely to develop metabolic disorders, while patients with metabolic dysfunctions were more easily in a chronic low-grade inflammatory status [24]. Therefore, combined obesity-related metabolic morbidity and adipose tissue-mediated immune dysfunction had an extremely critical impact on the survival of severe infectious patients with BSIs attributed to CRKP. Meanwhile, possibly excessive adipose tissue including VAT and SAT served as reservoirs for microorganisms such as *Mycobacterium tuberculosis*, HIV, influenza A virus, coronavirus according to previous research [24, 25].

Our results demonstrated that high total adipose tissue is independently associated with worse clinical outcomes, after adjusting for comorbid conditions and other differences in baseline characteristics. According to an in *vitro* analysis of two different human adipose tissues (VAT and SAT), VAT was likely implicated in the production of more proinflammatory cytokines, such as interleukin-6(IL-6), interleukin-8(IL-8), tumor necrosis factor-α (TNF-α), monocyte chemoattractant protein-1(MCP-1) [26]. Nevertheless, in our study based on the data analysis, VAT did not provide an important survival benefit in CRKP bloodstream infectious patients like COVID-19 in previously published studies [11, 20, 27]. We speculated that one of the reasons was attributed to Chinese people having a low BMI (lesser visceral adiposity) than European and American country individuals, so that VAT was not a particularly large proportion. Hence, further high-quality relative researches in this area are extremely crucial to verify this result in the future.

Besides, based on the multivariable analysis, the SOFA scores served to monitor daily organ dysfunction, and age were also significant indicators of risk factors for these severe difficult-to-treat infections. SOFA scores were considered as an effective and applicable prediction in-hospital all-cause mortality among infectious patients caused by multidrug-resistant Enterobacterales [28]. The higher of SOFA scores, the more organ (respiratory, renal, neurological, renal, and cardiovascular) dysfunction, which contributed to the increase of mortality and was not optimistic for the patient's prognosis [29].

There were several limitations in our work that must be acknowledged. One of our shortcomings was that the sample size was relatively small, which might limit the power of the research. Additionally, advanced age or multiple severe comorbidities potentially leads to worse clinical outcomes and increased risk of all-cause mortality among some of these patients. It was impossible to calculate the body surface area due to the lack of bodyweight that could not compare the density of muscle, visceral fat, and subcutaneous fat with the prognosis of infected patients. Besides, considering that CT scan is an expensive tool and has side effects, it may find obstacles that in clinical practices for CRKP BSI patients, there’s no need to perform abdominopelvic CT as a diagnostic tool. In spite of the above limitations, this was the first article to explore the relationship between CT-qualified components and mortality among patients who suffered from CRKP bloodstream infection. Although our study has some shortcomings, our results provided physicians with clinical significance for the association between body components and prognosis of patients with CRKP bloodstream infection. In addition, the nomogram of 14-day and 30-day mortality in BSI of CRKP can assist clinicians to judge the prognosis of these crucial infectious individuals and take some effective interventions to increase survival at an early time. Further high-quality prospective researches in this area are extremely needed in the future.

## Conclusion

Our study suggested that CT-derived body composition could be a credible and effective alternative to assess the prognosis of patients with BSI owing to CRKP. CT-quantified total adipose tissue, age, and SOFA scores were independently associated with 30-day all-cause mortality in these severe infectious patients, while skeletal muscle did not have obvious statistical significance.

## Data Availability

The dataset used and/or analyzed during the current study are available from the corresponding author on reasonable request.

## References

[1] Li Y, Sun QL, Shen Y, Zhang Y, Yang JW, Shu LB, Zhou HW, Wang Y, Wang B, Zhang R (2018). Rapid increase in prevalence of carbapenem-resistant enterobacteriaceae (CRE) and emergence of colistin resistance gene mcr-1 in CRE in a hospital in Henan, China. J Clin Microbiol.

[2] Cassini A, Högberg LD, Plachouras D, Quattrocchi A, Hoxha A, Simonsen GS, Colomb-Cotinat M, Kretzschmar ME, Devleesschauwer B, Cecchini M (2019). Attributable deaths and disability-adjusted life-years caused by infections with antibiotic-resistant bacteria in the EU and the European Economic Area in 2015: a population-level modelling analysis. Lancet Infect Dis.

[3] Shen L, Lian C, Zhu B, Yao Y, Yang Q, Zhou J, Zhou H (2020). Bloodstream infections due to carbapenem-resistant klebsiella pneumoniae: a single-center retrospective study on risk factors and therapy options. Microb Drug Resist.

[4] Tumbarello M, Trecarichi EM, De Rosa FG, Giannella M, Giacobbe DR, Bassetti M, Losito AR, Bartoletti M, Del Bono V, Corcione S (2015). Infections caused by KPC-producing Klebsiella pneumoniae: differences in therapy and mortality in a multicentre study. J Antimicrob Chemother.

[5] Gomez-Simmonds A, Nelson B, Eiras DP, Loo A, Jenkins SG, Whittier S, Calfee DP, Satlin MJ, Kubin CJ, Furuya EY (2016). Combination regimens for treatment of carbapenem-resistant Klebsiella pneumoniae bloodstream infections. Antimicrob Agents Chemother.

[6] Brink AJ (2019). Epidemiology of carbapenem-resistant Gram-negative infections globally. Curr Opin Infect Dis.

[7] Akahori D, Suzuki Y, Yokomura K, Shirai M, Yasui H, Hozumi H, Karayama M, Furuhashi K, Enomoto N, Fujisawa T (2019). Body composition changes successfully classify prognosis in patients with mycobacterium avium complex lung disease. J Infect.

[8] Huang Y, Lu Y, Huang YM, Wang M, Ling W, Sui Y, Zhao HL (2020). Obesity in patients with COVID-19: a systematic review and meta-analysis. Metabolism.

[9] Stanley TL, Feldpausch MN, Oh J, Branch KL, Lee H, Torriani M, Grinspoon SK (2014). Effect of tesamorelin on visceral fat and liver fat in HIV-infected patients with abdominal fat accumulation: a randomized clinical trial. JAMA.

[10] Malavazos AE, Corsi Romanelli MM, Bandera F, Iacobellis G (2020). Targeting the adipose tissue in COVID-19. Obesity (Silver Spring).

[11] Petersen A, Bressem K, Albrecht J, Thiess HM, Vahldiek J, Hamm B, Makowski MR, Niehues A, Niehues SM, Adams LC (2020). The role of visceral adiposity in the severity of COVID-19: highlights from a unicenter cross-sectional pilot study in Germany. Metabolism.

[12] Humphries RM, Ambler J, Mitchell SL, Castanheira M, Dingle T, Hindler JA, Koeth L, Sei K (2018). CLSI methods development and standardization working group best practices for evaluation of antimicrobial susceptibility tests. J Clin Microbiol.

[13] Ismail B, Shafei MN, Harun A, Ali S, Omar M, Deris ZZ (2018). Predictors of polymyxin B treatment failure in Gram-negative healthcare-associated infections among critically ill patients. J Microbiol Immunol Infect.

[14] Papst L, Beovic B, Pulcini C, Durante-Mangoni E, Rodriguez-Bano J, Kaye KS, Daikos GL, Raka L, Paul M, Esgap EE (2018). Antibiotic treatment of infections caused by carbapenem-resistant Gram-negative bacilli: an international ESCMID cross-sectional survey among infectious diseases specialists practicing in large hospitals. Clin Microbiol Infect.

[15] Stewardson AJ, Marimuthu K, Sengupta S, Allignol A, El-Bouseary M, Carvalho MJ, Hassan B, Delgado-Ramirez MA, Arora A, Bagga R (2019). Effect of carbapenem resistance on outcomes of bloodstream infection caused by Enterobacteriaceae in low-income and middle-income countries (PANORAMA): a multinational prospective cohort study. Lancet Infect Dis.

[16] Falcone M, Bassetti M, Tiseo G, Giordano C, Nencini E, Russo A, Graziano E, Tagliaferri E, Leonildi A, Barnini S (2020). Time to appropriate antibiotic therapy is a predictor of outcome in patients with bloodstream infection caused by KPC-producing *Klebsiella pneumoniae*. Crit Care.

[17] Nioche C, Orlhac F, Boughdad S, Reuzé S, Goya-Outi J, Robert C, Pellot-Barakat C, Soussan M, Frouin F, Buvat I (2018). LIFEx: a freeware for radiomic feature calculation in multimodality imaging to accelerate advances in the characterization of tumor heterogeneity. Cancer Res.

[18] Ying P, Jin W, Wu X, Cai W, Xia Y (2021). Association between CT-quantified body composition and recurrence, survival in nonmetastasis colorectal cancer patients underwent regular chemotherapy after surgery. Biomed Res Int.

[19] De Lorenzo A, Tarsitano MG, Falcone C, Di Renzo L, Romano L, Macheda S, Ferrarelli A, Labate D, Tescione M, Bilotta F (2020). Fat mass affects nutritional status of ICU COVID-19 patients. J Transl Med.

[20] Watanabe M, Caruso D, Tuccinardi D, Risi R, Zerunian M, Polici M, Pucciarelli F, Tarallo M, Strigari L, Manfrini S (2020). Visceral fat shows the strongest association with the need of intensive care in patients with COVID-19. Metabolism.

[21] Mitsou EK, Detopoulou M, Kakali A, Fragopoulou E, Nomikos T, Antonopoulou S, Panagiotakos DB, Kyriacou A (2019). Mining possible associations of faecal *A. muciniphila* colonisation patterns with host adiposity and cardiometabolic markers in an adult population. Benef Microbes.

[22] Leung CH, Liu CP (2019). Diabetic status and the relationship of blood glucose to mortality in adults with carbapenem-resistant *Acinetobacter baumannii* complex bacteremia. J Microbiol Immunol Infect.

[23] Iacobellis G, Penaherrera CA, Bermudez LE, Bernal Mizrachi E (2020). Admission hyperglycemia and radiological findings of SARS-CoV2 in patients with and without diabetes. Diabetes Res Clin Pract.

[24] Farzaei MH, Singh AK, Kumar R, et al. Targeting inflammation by flavonoids: novel: therapeutic strategy for metabolic disorders. Int J Mol Sci. 2019;20(19):4957. 10.3390/ijms20194957. 10.3390/ijms20194957 PMC6801776 31597283

[25] Lake JE, Debroy P, Ng D, Erlandson KM, Kingsley LA, Palella FJ, Budoff MJ, Post WS, Brown TT (2019). Associations between subcutaneous fat density and systemic inflammation differ by HIV serostatus and are independent of fat quantity. Eur J Endocrinol.

[26] Fain JN (2006). Release of interleukins and other inflammatory cytokines by human adipose tissue is enhanced in obesity and primarily due to the nonfat cells. Vitam Horm.

[27] Battisti S, Pedone C, Napoli N, Russo E, Agnoletti V, Nigra SG, Dengo C, Mughetti M, Conte C, Pozzilli P (2020). Computed tomography highlights increased visceral adiposity associated with critical illness in COVID-19. Diabetes Care.

[28] Kurtz P, Taccone FS, Bozza FA, et al. Systemic severity and organ dysfunction in subarachnoid hemorrhage: a large retrospective multicenter cohort study. Neurocrit Care. 2021;35(1):56–61. 10.1007/s12028-020-01139-3. 10.1007/s12028-020-01139-3 33150574

[29] Antunes ML, Seixas J, Ferreira HE, Silva MS. Adequacy of severe malaria markers and prognostic scores in an intensive care unit in Luanda, Angola: a clinical study. J Clin Med. 2020;9(12):3862. 10.3390/jcm9123862. 10.3390/jcm9123862 PMC7760046 33261096

